# Development of Automated Risk Stratification for Sporadic Odontogenic Keratocyst Whole Slide Images with an Attention-Based Image Sequence Analyzer

**DOI:** 10.3390/diagnostics13233539

**Published:** 2023-11-27

**Authors:** Samahit Mohanty, Divya B. Shivanna, Roopa S. Rao, Madhusudan Astekar, Chetana Chandrashekar, Raghu Radhakrishnan, Shylaja Sanjeevareddygari, Vijayalakshmi Kotrashetti, Prashant Kumar

**Affiliations:** 1 Department of Computer Science and Engineering, M. S. Ramaiah University of Applied Sciences, Bengaluru 560054, India; samahit@gmail.com; 2 Department of Oral Pathology and Microbiology, Faculty of Dental Sciences, M. S. Ramaiah University of Applied Sciences, Bengaluru 560054, India; 3 Department of Oral Pathology, Institute of Dental Sciences, Bareilly 243006, India; madhutanu@gmail.com; 4 Department of Oral & Maxillofacial Pathology & Microbiology, Manipal College of Dental Sciences, Manipal 576104, India; chetana.c@manipal.edu (C.C.); raghu.ar@manipal.edu (R.R.); 5 Department of Oral Pathology, SVS Institute of Dental Sciences, Mahbubnagar 509001, India; sailajasanjeeva@gmail.com; 6 Department of Oral & Maxillofacial Pathology & Microbiology, Maratha Mandal’s Nathajirao G Halgekar, Institute of Dental Science & Research Centre, Belgaum 590010, India; drviju18@yahoo.com; 7 Department of Oral & Maxillofacial Pathology, Nijalingappa Institute of Dental Science & Research, Gulbarga 585105, India; munna.pmk@gmail.com

**Keywords:** deep learning, recurring OKC, non-recurring OKC, whole slide imaging, vision transformer, image classification

## Abstract

(1) Background: The categorization of recurrent and non-recurrent odontogenic keratocyst is complex and challenging for both clinicians and pathologists. What sets this cyst apart is its aggressive nature and high likelihood of recurrence. Despite identifying various predictive clinical/radiological/histopathological parameters, clinicians still face difficulties in therapeutic management due to its inherent aggressive nature. This research aims to build a pipeline system that accurately detects recurring and non-recurring OKC. (2) Objective: To automate the risk stratification of OKCs as recurring or non-recurring based on whole slide images (WSIs) using an attention-based image sequence analyzer (ABISA). (3) Materials and methods: The presented architecture combines transformer-based self-attention mechanisms with sequential modeling using LSTM (long short-term memory) to predict the class label. This architecture leverages self-attention to capture spatial dependencies in image patches and LSTM to capture sequential dependencies across patches or frames, making it suitable for this image analysis. These two powerful combinations were integrated and applied on a custom dataset of 48 labeled WSIs (508 tiled images) generated from the highest zoom level WSI. (4) Results: The proposed ABISA algorithm attained 0.98, 1.0, and 0.98 testing accuracy, recall, and area under the curve, respectively, whereas VGG16, VGG19, and Inception V3, standard vision transformer attained testing accuracies of 0.80, 0.73, 0.82, 0.91, respectively. ABISA used 58% fewer trainable parameters than the standard vision transformer. (5) Conclusions: The proposed novel ABISA algorithm was integrated into a risk stratification pipeline to automate the detection of recurring OKC significantly faster, thus allowing the pathologist to define risk stratification faster.

## 1. Introduction

OKCs account for 3–11% of all jaw cysts. They are benign neoplasms of odontogenic origin recognized for their invasive tendency. Of all odontogenic cysts, OKCs are of great interest in terms of high propensity to recur after surgical treatment, making up (2.5%, 2–100%). These significant differences are due to various postoperative follow-up periods, the operational methods used, or the inclusion of Nevoid basal cell carcinoma syndrome (NBCCS) cases [1,2].

It is essential to note that the distinction between recurring and non-recurring OKCs can sometimes be challenging based on clinical and radiographic features alone. Other factors, such as the specific location of the cyst, patient factors, and genetic factors, may also influence the likelihood of recurrence. A close follow-up, evidenced by histopathology confirmation, is essential in managing OKCs to monitor recurrence and ensure appropriate treatment [3].

Per the literature evidence, various histological determinants predicting recurrence are para keratinization, basal mitosis, subepithelial split, satellite cysts, dental lamina rests, basal cell budding, reverse polarity, dense collagen, and diffuse inflammation and recently added additional histopathological (h/p) features that strongly suggest recurrence to the existing list including subepithelial hyalinization, incomplete cystic lining, and the corrugated/wavy surface [4,5,6].

Patch-level classification results in a better classification of the entire slide image after aggregating patch-level results with a fusion model. This is performed with the output resulting from CNN on patches. An expectation maximization (EM) technique automatically identifies discerning patches with resilience, utilizing the spatial connections among these patches [7].

Automating whole slide classification on cancer images is quite common. A lower zoom level is recommended to detect artifacts, tissue areas, and abnormalities. WSIs can be stored for years to refer to different results. Automation can help minimize diagnosis errors or help pathologists obtain first-hand opinions. Identifying the relevant areas of interest inside WSIs becomes critical for the algorithm to succeed. VIT performs better than CNN when considering a higher resolution of WSIs [8]. 

Manual analysis of the whole slide image is time-consuming. The availability and accessibility of powerful computers make it easy to automate the detection of different diseases with automated systems. The issue with the current computer-aided system is the availability of standard datasets with the right annotations. CNN may fail to perform feature extraction on specific areas of interest in the whole slide image. Hence, the attention mechanism is appropriate for integration with CNN to achieve better performance in such cases [9].

This study aims to develop an automation system that classifies whole slide images as either recurring OKC or non-recurring OKC based on specific h/p features. The current study is the first to analyze recurring and non-recurring OKC using whole slide images [10].

Also called a digital or virtual slide, a WSI is a high-resolution digital rendering of an entire histopathology glass slide encapsulated in gigabytes of data. This technology captures the comprehensive image in a single sweep, affording the capability to zoom in and out on regions of interest (ROIs), an otherwise arduous task when using microscopy. The resultant digital depiction is an expansive, multi-gigapixel file, meticulously conserving all the inherent data within the original glass slide. This WSI platform opens the door to diverse image analysis methodologies, encompassing computer-aided algorithms for quantifying and extracting features [11].

## 2. Related Work

### 2.1. Related Work on Whole Slide Image Challenges

The successful utilization of deep learning in analyzing whole slide images (WSIs) holds the potential to develop advanced clinical tools that excel in accuracy, reproducibility, and impartiality compared with current clinical methods. This approach also offers fresh insights into various pathological conditions. However, WSIs are large, multi-gigabyte images with resolutions of around 100,000 × 100,000 pixels. Existing hardware struggles to accommodate learning from such high-resolution images, necessitating some form of dimensionality reduction [12].

### 2.2. Related Work on Preprocessing Images and Class Imbalances

In a previous study, whole slide images were taken from The Cancer Genome Atlas (TCGA) dataset and stained using hematoxylin and eosin. All the images with the highest resolution were taken and resized. A tile size of 1024 × 1024 was performed at 20× resolution. A trained pathologist investigated all these tiles to label them. Tiles with less information were discarded [13].

Generative adversarial networks (GANs) are very good at generating synthetic data that keep the probability distribution of the original data intact. GANs can be used to create artificial data that are statistically similar to actual data. A hybrid system with GAN often solves the class imbalance problem. Class imbalance can lead to poor performance, as the recommendation system may learn to favor the majority class. A hybrid GAN approach addresses this problem by generating synthetic negative examples to balance the dataset. It helps to improve the performance of the recommendation system. In one study, GANs were trained using an adversarial setting, where the generator and discriminator constantly tried to outsmart each other. This process forced the generator to learn to produce increasingly realistic data while the discriminator learned to become better at differentiating between real and fake data [14].

### 2.3. Related Work on Vision Transformer in Image Processing

The advancements achieved using transformer networks in natural language processing have sparked significant interest among the computer vision community to apply these models to vision-related tasks. These fundamental concepts played a role in the development of traditional transformer models. The concept of self-attention enables capturing “long-term” connections between elements within a sequence, a capability lacking in conventional recurrent models that struggle to encode such associations [15]. 

The vision transformer (ViT) stands out as a trailblazer in demonstrating that a pure transformer architecture can achieve exceptional performance comparable to models like ResNets and EfficientNet in image classification tasks. This accomplishment becomes evident when dealing with sufficiently large datasets like ImageNet-22k and JFT-300M. ViT’s methodology involves partitioning each image into sequences of fixed-length tokens (non-overlapping patches) and subsequently using standard transformer layers, which encompass both the multi-head self-attention module (MHSA) and the position-wise feed-forward module (FFN), to examine and depict these tokens [16].

### 2.4. Related Work on Deep Learning in OKC

In a previous study, the multi-model ensemble learning technique delivered satisfactory outcomes when distinguishing between recurrent and non-recurrent categories of OKCs. Additionally, the predictions generated individually with classifiers and using the conventional ensemble method displayed effectiveness, achieving an accuracy spanning from 85% to 93% in categorizing the dataset. In this study, the ensemble model was outperformed by other evaluated models, including the traditional ensemble [17].

## 3. Materials and Methods

This section elaborates on preprocessing and the proposed algorithm with a flow diagram. 

### 3.1. Data Collection

A collaborative study encompassed the pooling of slide archives from multiple centers, resulting in the consolidation of 48 histopathology slides from 113 reported between 2015 and 2020.

The Faculty of Dental Sciences, MSRUAS, Manipal College of Dental Sciences (MCODS) in Manipal, Institute of Dental Science in Bareilly, S Nijalingappa Institute of Dental Sciences and Research in Rajapur, Gulbarga, Karnataka, Maratha Mandal’s Nathajirao G Halgekar, Institute of Dental Science & Research Centre in Belgaum, Karnataka, and the SVS Institute of Dental Sciences in Mahbubnagar, Andhra Pradesh, India, voluntarily participated in the research initiative. Ethics approval was waived due to the retrospective nature of this study, and the collected slides were anonymized and encoded to eliminate any patient identifiers by other centers. Our institute permitted this study with ethics clearance (NO.EC-2021/F/058) for the archived slides. Based on the features identified in the pilot study by Augustine, D., Rao, R. S et al. (2021) [18], histo/pathology features were re-evaluated on H and E-stained slides and recorded using WSI [18]. This study included slides with no artifacts, good staining quality, and complete clinical records with post-treatment follow-up. 

The slides were digitalized using a whole slide imaging scanner (Morphle Labs Whole Slide Scanner Model INDEX). A total of 48 OKC whole slide images were collected with various zoom levels. Slide sizes vary from 50 megabytes to 3 gigabytes depending on the scanner and zoom level used to obtain the whole slide image. Out of these, 17 recurring and 31 non-recurring slides were identified by expert pathologists.

### 3.2. Data Preprocessing and Dataset Generation

**Figure 1:**
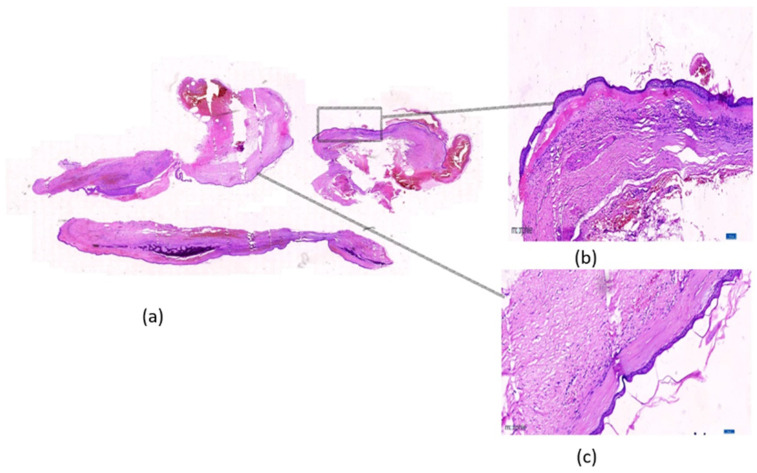
The whole slide image shows recurring-odontogenic keratocyst with subepithelial hyalinization (high risk). (**a**) Scanner view of recurrent Odontogenic Keratocyst Hematoxylin and Eosin-stained whole slide image, Magnification 1.3×; (**b**) Recurrent Odontogenic Keratocyst with surface corrugation, subepithelial hyalinization, and incomplete epithelial lining. Magnification 10×; (**c**) Band of subepithelial hyalinization and surface corrugation. Magnification 20×.

**Figure 2:**
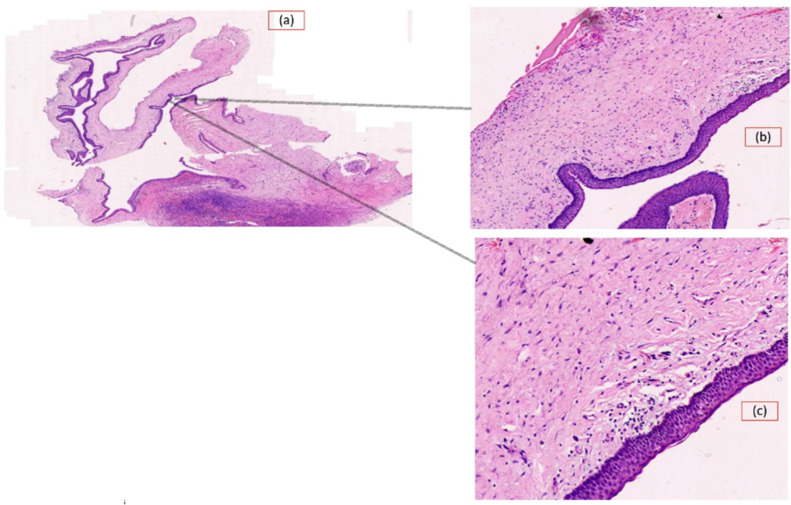
The whole slide image is non-recurring- odontogenic keratocyst (low risk). (**a**) Scanner view of Hematoxylin and Eosin-stained whole slide image Non-recurrent Odontogenic Keratocyst, magnification 1.3×; (**b**) Non-recurrent Odontogenic Keratocyst. Absence of subepithelial hyalinization. Magnification 10×; (**c**) Absence of subepithelial hyalinization is further enhanced. Magnification 20×.

**Figure 3:**
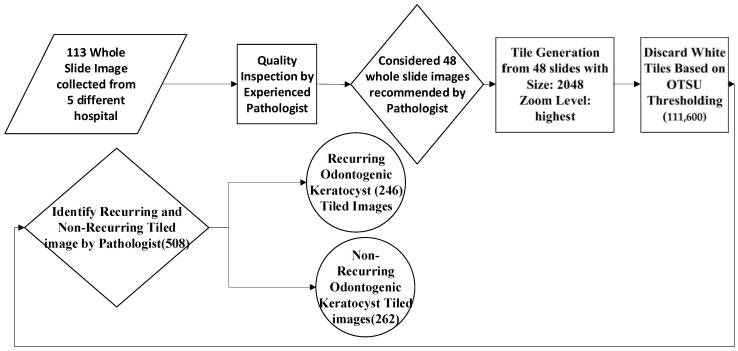
Dataset generation process flow diagram.

A total of 48 of the 113 OKC WSIs (e.g., Figure 1 and Figure 2) were identified for this purpose and evaluated by a pathologist to segregate and annotate as recurring and non-recurring slides. An experienced pathologist was engaged in annotating the slides. To rule out subjectivity and inter-observer bias, a third pathologist, who was part of this study, was consulted, and the pathologists arrived at a consensus. Post-annotation, these slides were processed using an automated tile image generation system developed based on an open-source library open slide and deep zoom generator. This automated system generated a tiled image size of 2048 × 2048 and discarded white tiles. The same was performed by calculating the entropy and variance in each pixel value in each tile. Tiles with very low entropy or variance were likely to contain uniform or low information, so these were considered empty tiles or white tiles. After this, each of them was inspected by the pathologist again and was correctly labeled as recurring or non-recurring. The highest zoom-level slides were considered to generate the tiles during this preprocessing, as shown in Figure 3. The entire dataset generation process flow is shown in Figure 3.

### 3.3. Attention-Based Image Sequence Analyzer

**Figure 4:**
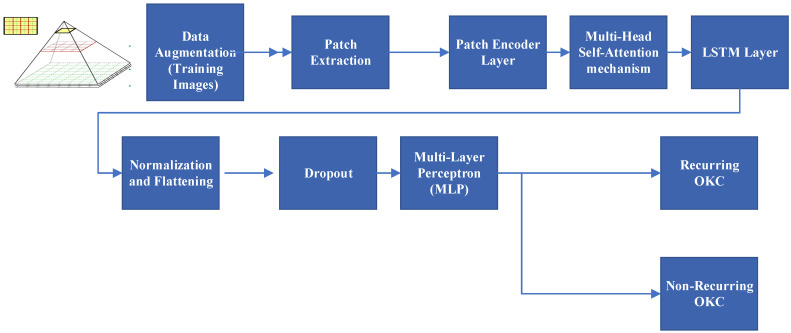
Attention-based image sequence analyzer algorithm flow for recurring and non-recurring OKC.

The attention-based image sequence analyzer (ABISA) architecture is a hybrid model combining multi-head self-attention-based transformer architecture elements with the LSTM layer. The LSTM layer is used to capture temporal dependencies. This combination leverages the spatial relationships captured by the self-attention mechanism and enhances it with LSTM’s ability to model sequential patterns. The architecture flow diagram is described in Figure 4.

### 3.4. Image Data Augmentation

Using the Keras image data generator, preprocessed tiles with the appropriate label were processed with the following parameters for the augmentation of images during the training process. A 70-10-20 rule for training, validation, and testing was used, i.e., 20% of the tiled images from different classes were used for testing the classification model. The following values were used for augmentation: rotation range = 20, width_shift_range = 0.1, height_shift_range = 0.1, shear range = 0.2, zoom_range = 0.2, and horizontal flip = True.

### 3.5. Patch Extraction

The patches extraction layer is a custom Keras layer that takes an input image and extracts non-overlapping patches. This process allows the model to process smaller image regions independently, providing spatial invariance and reducing computational complexity. For the patch size used in model was 6, each input image was divided into non-overlapping patches of size 6 × 6 pixels. The calculation num_patches = (image_size // patch_size) ** 2 is used to determine the total number of patches extracted from the input images. In this model, for an image with dimensions 64 × 64 (image_size = 64) and extracted patches of size 6 × 6, (64 // 6) ** 2 is used to calculate the total number of patches: 100. It extracted 100 patches from the 64 × 64 input image, each of size 6 × 6 pixels. The patches layer and the subsequent processing extracted and processed these patches for further transformation and classification.

### 3.6. Patch Encoder Layer

The patch encoder layer takes the extracted patches and encodes them into a meaningful representation. It uses dense (fully connected) layers and an embedding layer to map the patches into a higher-dimensional space. Additionally, it incorporates positional embeddings to retain spatial information about the original image patches. projection_dim = 64 is used in this model. This value specifies the dimensionality of the embeddings generated for each patch, which are projected to a 64-dimensional space. These encoded patch representations are then used in subsequent layers of the ABISA model to perform tasks like self-attention and classification.

### 3.7. Multi-Head Self-Attention Mechanism

A multi-head self-attention mechanism is used to analyze the input patches (encoded patches). It captures complex relationships and dependencies among them. The multi-head attention layer involves three key parameters. Those are key dimensions, values, and queries.

The key dimension parameter determines the number of attention heads used in parallel. Each attention head specializes in learning distinct aspects of the relationships between input patches. Notably, within each attention head, the dimensionality defined by the key dimension is consistently applied to queries, keys, and values; all are set to 64 in this case.

A dropout rate is applied to the attention scores to facilitate regularization. This means that the model computes attention scores to determine how each element in the encoded patches attends to every other element within itself, known as self-attention. Based on the relationships and interactions between the patches, the multi-head self-attention mechanism operation creates the attention output.

Skip Connection and Layer Normalization: After the attention operation, the attention output is combined with the original encoded patches using a skip connection (element-wise addition). Layer normalization is applied to the combined output to ensure stable training and to help with gradient flow during training.

### 3.8. LSTM Layer

**Figure 5:**
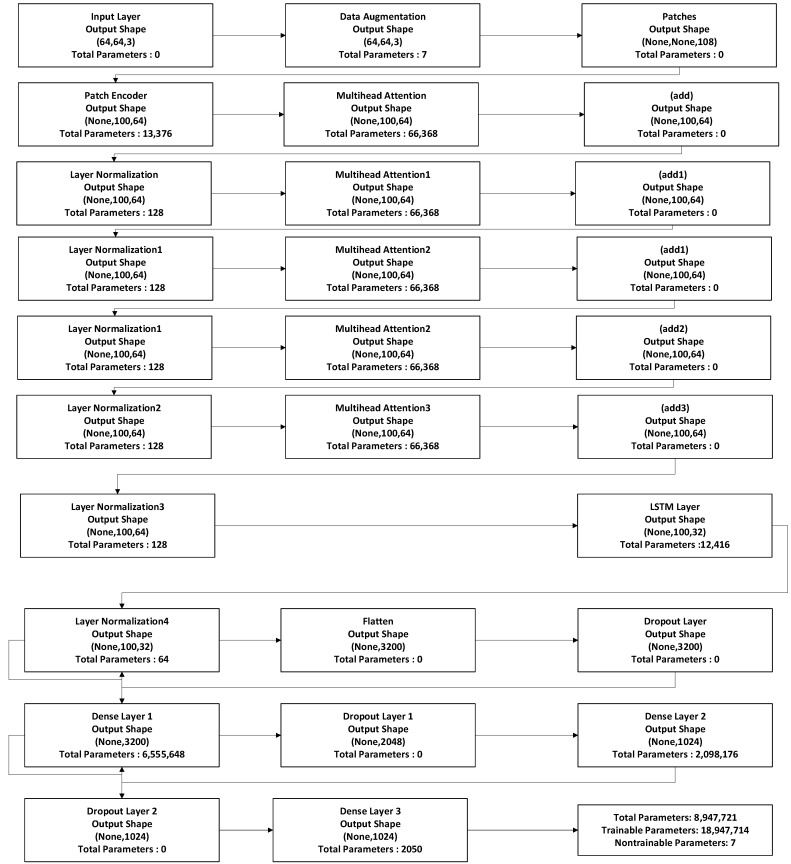
Model summary of the attention-based image sequence analyzer.

The proposed LSTM layer had 32 units in its hidden and cell states. The encoded patches with attention were passed through the LSTM layer, which was designed to process sequential data. The LSTM layer takes the encoded patches with attention as input and processes them sequentially, considering the temporal order of the patches. This LSTM layer performs sequence modeling and captures temporal dependencies in the data. The output is a sequence of feature vectors representing the input data’s processed information, incorporating both spatial and sequential information. Figure 5 describes model summary of proposed model.

### 3.9. Normalization and Flattening

The LSTM output was normalized and flattened to prepare it for further processing.

### 3.10. Dropout

The dropout layer helps prevent overfitting, in this case, 0.5 or 50%. This helps reduce reliance on specific features and encourages the model to learn more robust representations.

### 3.11. Multi-Layer Perceptron (MLP)

The GELU (Gaussian Error Linear Unit) activation function was used in the classification layer to predict the output.

## 4. Results

The dataset was experimented on using the standard CNN, pre-trained models, and vision transformer algorithms for image classification. The proposed model, which classifies recurring and non-recurring OKC, is better for the given dataset. Since the proposed model extends standard state-of-the-art vision transformer architecture, a comparison was made between the proposed model and the standard vision transformer model in the following section. Table 1 describes the overall results of the performance metrics of various experiment models. Table 2 gives all the hyperparameters used in the proposed model.

### 4.1. Confusion Matrix

**Figure 6:**
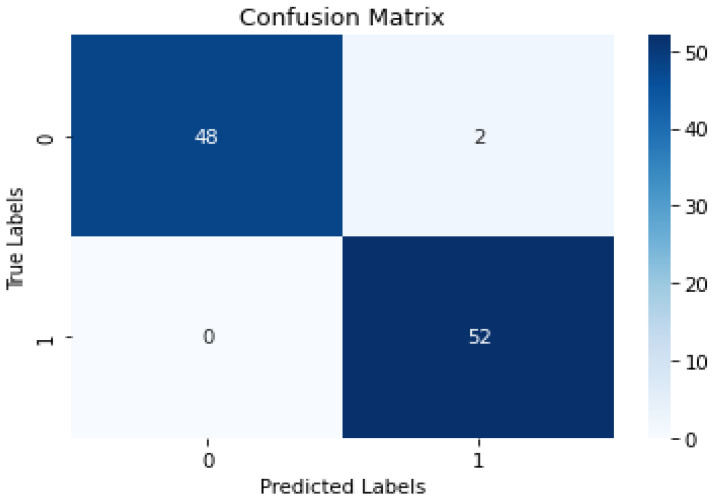
Attention-based image sequence analyzer confusion matrix.

A confusion matrix is a 2 × 2 table used in classification tasks to assess the performance of a machine learning model, as shown in Figure 6 below.

### 4.2. ROC (Receiver Operating Characteristic) Curve

The area under the ROC curve (AUC) is a standard metric; a higher AUC indicates better model discrimination ability. The ROC is used to evaluate the performance of classification models. It plots the true positive rate (sensitivity) against the false positive rate (1-specificity) at various classification thresholds. The ROC curve illustrates how well the model distinguishes between positive and negative classes. The AUC score is a useful metric in situations where class imbalances exist in the dataset. It also provides a single value to compare different classifiers’ performance, making evaluating and choosing the best model for a given task easier. The score of 0.98 indicates the classifier’s performance is better compared with the standard ViT’s score of 0.94.

**Figure 7:**
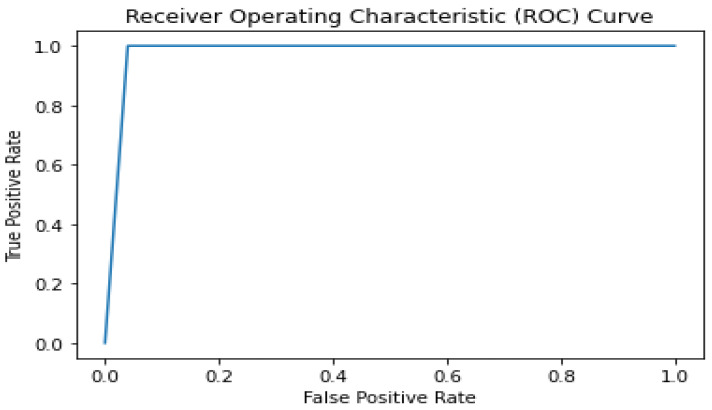
Attention-based image sequence analyzer ROC curve.

The ROC curve is given in Figure 7 for the proposed model.

### 4.3. Training vs. Validation Loss Curve

The training vs. validation loss curve is a plot that shows the changes in the training and validation loss during the training process of a machine learning or deep learning model.

**Figure 8:**
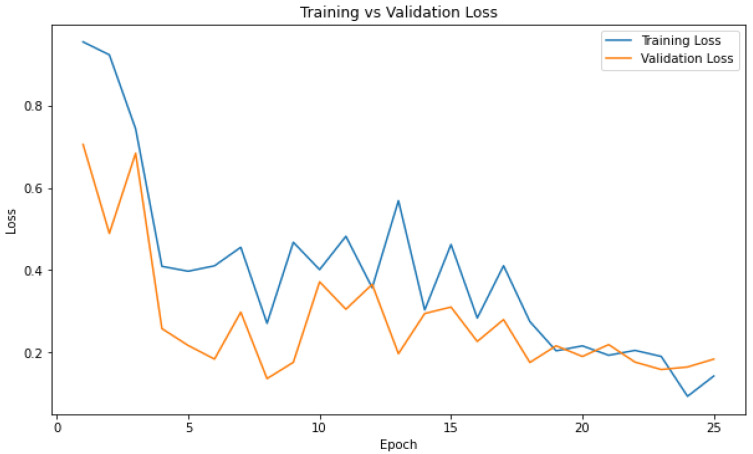
Attention-based image sequence analyzer training vs. validation.

The training vs. validation loss curve was plotted with the epochs (training iterations) on the *x*-axis and the corresponding loss values on the *y*-axis. As the model was trained over multiple epochs, the training loss generally decreased because the model was learning to fit the training data better. However, the validation loss might behave differently. Initially, it decreased along with the training loss as the model generalized better. However, at some point, the validation loss started to increase. This indicates that the model was overfitting the training data, and its performance on the validation data was deteriorating, even though it improved on the training data. So, training should be stopped at this epoch, which is the 24th epoch in this study. Figure 8 shows the curve.

### 4.4. Classification Report—ABISA

A classification report summarizes performance metrics for a classification model, typically presented in a tabular format. It includes key metrics such as precision, recall, F1-score, and accuracy for each class in a classification problem. This report provides insights into the model’s performance for individual classes, highlighting strengths and weaknesses. It is a valuable tool for evaluating the effectiveness of a classification model across different categories. Table 3 describes the classification report for the proposed model.

### 4.5. Log Loss 

Log loss (logarithmic loss) is a commonly used loss function for evaluating the accuracy of probabilistic classification models, such as logistic regression or neural networks, that predict probabilities for each class. It measures the discrepancy between predicted probabilities and target values, penalizing more significant deviations. Lower log loss values indicate better alignment between predicted probabilities and actual outcomes. Table 4 describes a comparison of log loss among the models.

The proposed model has a log loss value of 0.13, indicating that the model’s predicted probabilities are pretty accurate and very close to the true labels. A log loss close to zero in binary classification indicates excellent performance, as the model’s predicted probabilities align well with the actual outcomes.

A standard ViT log loss value of 1.04 means that, on average, the model’s predicted probabilities are slightly far from the true labels. The model’s prediction confidence might be relatively low compared with the proposed model. Table 5 describes different metrics for the proposed model.

### 4.6. Pipeline Result

**Figure 9:**
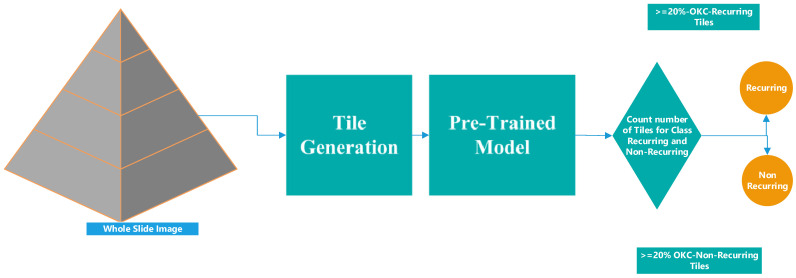
Workflow diagram of pipeline WSI prediction.

The devised pipeline system aimed at forecasting whether an entire whole slide image (WSI) corresponds to recurring or non-recurring OKC instances. This pipeline system takes WSI as input, tiles the WSI in the pre-processing step, and then applies the proposed attention-based image sequence analyzer to classify the tiles as the recurring class or not. Based on the counts of predicted tiles in both classes, a designated threshold was formulated. This threshold was established considering factors like slide size and zoom level. While a 15% threshold was typically effective, smaller-sized slides (up to 500 megabytes) might necessitate a lower threshold. The threshold value was determined by collaborating with a pathologist with comprehensive knowledge of the slide scanner. To elaborate, this approach was tested across seven distinct slides, yielding the subsequent statistical outcomes. The pipeline testing considered a total of seven (four non-recurring and three recurring) WSIs (H and E-stained and scanned recurrent and non-recurrent WSIs); these seven WSIs were not used during the training and validation of the model. The prognostic accuracy of the framework in the presence of a pathologist was independently reported. Therefore, this model can be clinically applied to classify recurrent and non-recurrent OKCs. Figure 9 describes the process flow. Table 6 describes the sample whole slide image taken for pipeline experiments.

## 5. Discussion

This study performed different experiments on both the proposed and all state-of-the-art algorithms available for whole-slide image processing. Due to the massive size of the whole slide image, it was essential to split the images into multiple smaller images called tiled images. These images have a size of 2048, which helped to visualize these images with a standard computer and label them correctly by an expert pathologist. This manual label was a slightly longer process as each tiled image was labeled. During the experiment on this custom-labeled dataset, training was performed using standard available models like CNN, VGG16, VGG19, and Inception V3. Various model hyperparameters were tuned repeatedly during the experiment, and multiple experiments were carried out. The objective was to have high classification accuracy. The proposed ABISA model was built on top of the vision transformer model. With the increasing application of the vision transformer model into computer vision, here, the VIT architecture was customized to suit the OKC histopathology image dataset. Introducing the LSTM layer with a size of 32 after the self-attention block reduced the dimension from 64 to 32. This dimension reduction contributed to a significant reduction in the number of parameters as OKC was recurring, and non-recurring OKC was of smaller feature space. This model was more computationally efficient. The model was more parameter-efficient by reducing the dimensionality of the features before feeding them into the dense layers. This was particularly useful as fewer images were considered for training. Multiple experiments were conducted to arrive at an LSTM layer value of 32. The LSTM layer’s integration helped maintain a memory of past states while processing new patches. This was crucial for recognizing patterns that span across multiple patches in an image. It can learn to recognize object shapes, contours, and other sequential patterns contributing to image classification. The LSTM was beneficial when dealing with sequences of images where temporal relationships played a crucial role. Overall, by including an LSTM layer, this architecture creates a hybrid model that capitalizes on spatial and temporal dependencies. This integration is beneficial when working with images containing sequential patterns or aiming for higher-level feature abstraction. By training those OKC images with complete clinical variables and follow-up, the possibility of bias is eliminated. The trained model classified the risk of recurrence into recurrent and non-recurrent OKCs based on histopathological evaluation. Further validation was performed by blinding the images pooled from other centers to rule out false positives and negatives, meeting clinical standards. 

## 6. Conclusions

The proposed pipeline for risk stratification of OKCs is a powerful tool to improve patients’ dental health. The proposed model significantly reduced (58%) trainable parameters compared with its peer state-of-the-art algorithms including vision transformer. A recall of 1.0 and precision of 0.96 suggested confidence in the proposed model for detecting recurring OKC correctly. These results stratified risks into correct groups of recurring or non-recurring OKC. This pipeline significantly boosts pathologists’ detection of recurring and non-recurring OKC. This model could be utilized locally or remotely based on any OKC WSI. The attention-based image sequence analyzer (ABISA) model reduces training time significantly and can detect an entire WSI in less than 10 min. Most of the time, the execution time depends on WSI size. The entire process was automated to use WSIs as input, concluding risk stratification for odontogenic keratocysts. Hence, managing such patients for dentists became comparatively smoother. 

## 7. Future Work

Although the proposed model has a very high accuracy of detecting recurring and non-recurring OKC at the tile level, slide-level accuracy depends on the number of tiles having features of recurring and non-recurring. The dataset ignores blurred images and discards images that are not adequately stained. This study could help clinicians plan surgical management well in advance based on the automated h/p report. This adds to the advantage of adopting a conservative mode of treatment in our institute. Transformer-based architecture is rapidly gaining popularity in computer vision applications. Hence, this research can be suitably extended to different cancers where whole slide images are the gold standard for detecting cancer. 

## Figures and Tables

**Table 1:** Comparison of model performance metrics and parameters.

MODEL	Recall	Precision	F1-Score	AUC	Accuracy	Total Parameters
Standard convolution neural network (CNN)	0.86	0.86	0.84	0.93	0.84	683,329
VGG19	0.73	0.81	0.73	0.77	0.73	131,585
VGG16	0.80	0.84	0.80	0.82	0.80	131,585
Inception V3	0.82	0.82	0.82	0.78	0.82	23,901,985
Standard ViT	0.95	0.86	0.90	0.91	0.91	15,488,969
Proposed attention-based image sequence analyzer	1.00	0.96	0.98	0.98	0.98	8,947,721

**Table 2:** Comparison of the model hyper-parameters with the standard VIT.

Component	ViT	Proposed Attention-Based Image Sequence Analyzer
learning_rate	0.0001	0.0001
Batch size	20	20
Epochs	25	25
weight_decay	0.001	0.001
patch_size	6	6
projection_dim	64	64
num_heads	4	4
transformer_layers	4	4
mlp_head_units	[2048, 1024]	[2048, 1024]
dropout_rate	0.1	0.1
lstm_units	NA	32
Optimizer	Adam	Adam
Loss function	SparseCategoricalCrossentropy	SparseCategoricalCrossentropy
Activation function	Gaussian Error Linear Unit	Gaussian Error Linear Unit

**Table 3:** Attention-based image sequence analyzer classification report.

	Precision	Recall	F1-Score	Support
0	1.00	0.96	0.98	50
1	0.96	1.00	0.98	52
Accuracy			0.98	102
Macro average	0.98	0.98	0.98	102
Weighted average	0.98	0.98	0.98	102

**Table 4:** Log loss comparison.

Model	Log Loss
Standard CNN	2.87
VGG19	9.72
VGG16	7.29
Inception V3	6.41
Standard ViT	1.04
Attention-based image sequence analyzer	0.13

**Table 5:** Performance metrics.

Metrics	Value
Accuracy	0.98
Precision	0.96
Recall	1.0
F1-score	0.98
Matthews correlation coefficient	0.96
Cohen’s kappa	0.96
Balanced accuracy	0.98
Jaccard score	0.96
Brier score loss	0.01
Specificity (true negative rate)	0.96
Sensitivity (true positive rate)	1.0
Youden’s index (J)	0.96
G-mean	0.97
Log loss	0.13

**Table 6:** Sample stats of pipeline result.

Case No.	File Size (MB)	Base Resolution (H, W)	No. of Tiles	No. of Recurring OKC Tiles	No. of Non-Recurring OKC Tiles	Predicted Output	Actual Output
HP 68/22	658.6	126,976 × 126,976	3844	751	3093	Recurring	Recurring
HP 86/22	793.3	126,982 × 126,982	4153	171	3982	Non- recurring	Non- recurring

## Data Availability

Restrictions apply to the availability of these data. Data was obtained from the mentioned centers and is available from the authors with permission.

## References

[1] Kuroyanagi N., Sakuma H., Miyabe S., Machida J., Kaetsu A., Yokoi M., Maeda H., Warnakulasuriya S., Nagao T., Shimozato K. (2009). Prognostic factors for keratocystic odontogenic tumor (odontogenic keratocyst): Analysis of clinicopathologic and immunohistochemical findings in cysts treated by enucleation. J. Oral Pathol. Med..

[2] Radhakrishnan R., Chandrashekar C., Patel P., Thennavan A. (2020). Odontogenic keratocyst: Analysis of recurrence by AgNOR, p53 and MDM2 profiling. J. Oral Maxillofac. Pathol..

[3] Diniz M.G., Borges R., Guimarães A.L.S., Moreira P.R., Brito J.A.R., Gomez M.V., De Marco L., Gomez R.S. (2009). PTCH1 isoforms in odontogenic keratocysts. Oral Oncol..

[4] Cottom H.E., Bshena F.I., Speight P.M., Craig G.T., Jones A.V. (2011). Histopathological features that predict the recurrence of odontogenic keratocysts. J. Oral Pathol. Med..

[5] Augustine D., Rao R.S., Patil S. (2021). Hyalinization as a histomorphological risk predictor in oral pathological lesions. J. Oral Biol. Craniofacial Res..

[6] Keshani F., Jahanshahi G., Mirkazemi Z., Mirkazemi H. (2023). Evaluating histopathological factors of predicting the recurrence rate of odontogenic keratocyst. Dent. Res. J..

[7] Hou L., Samaras D., Kurc T.M., Gao Y., Davis J.E., Saltz J.H. Patch-Based Convolutional Neural Network for Whole Slide Tissue Image Classification. Proceedings of the 2016 IEEE Conference on Computer Vision and Pattern Recognition (CVPR).

[8] Hossain S., Shahriar G.M., Syeed M.M.M., Uddin M.F., Hasan M., Shivam S., Advani S. (2023). Region of interest (ROI) selection using vision transformer for automatic analysis using whole slide images. Sci. Rep..

[9] Rashmi R., Prasad K., Udupa C.B.K. (2021). Breast histopathological image analysis using image processing techniques for diagnostic purposes: A methodological review. J. Med. Syst..

[10] Fidele N., Yueyu Z., Zhao Y., Tianfu W., Liu J., Sun Y., Liu B. (2019). Recurrence of odontogenic keratocysts and possible prognostic factors: Review of 455 patients. Med. Oral Patol. Oral Cir. Buccal.

[11] Aeffner F., Zarella M.D., Buchbinder N., Bui M.M., Goodman M.R., Hartman D.J., Lujan G.M., Molani M.A., Parwani A.V., Lillard K. (2019). Introduction to Digital Image Analysis in Whole-slide Imaging: A White Paper from the Digital Pathology Association. J. Pathol. Inform..

[12] Dimitriou N., Arandjelović O., Caie P.D. (2019). Deep Learning for Whole Slide Image Analysis: An Overview. Front. Med..

[13] Barker J., Hoogi A., Depeursinge A., Rubin D.L. (2016). Automated classification of brain tumor type in whole-slide digital pathology images using local representative tiles. Med. Image Anal..

[14] Shafqat W., Byun Y.-C. (2022). A Hybrid GAN-Based Approach to Solve Imbalanced Data Problem in Recommendation Systems. IEEE Access.

[15] Han K., Wang Y., Chen H., Chen X., Guo J., Liu Z., Tang Y., Xiao A., Xu C., Xu Y. (2022). A survey on vision transformer. IEEE Trans. Pattern Anal. Mach. Intell..

[16] Yuan L., Chen Y., Wang T., Yu W., Shi Y., Jiang Z.H., Tay F.E., Feng J., Yan S. Tokens-to-token vit: Training vision transformers from scratch on imagenet. Proceedings of the IEEE/CVF International Conference on Computer Vision.

[17] Rao R.S., Shivanna D.B., Lakshminarayana S., Mahadevpur K.S., Alhazmi Y.A., Bakri M.M.H., Alharbi H.S., Alzahrani K.J., Alsharif K.F., Banjer H.J. (2022). Ensemble Deep-Learning-Based Prognostic and Prediction for Recurrence of Sporadic Odontogenic Keratocysts on Hematoxylin and Eosin Stained Pathological Images of Incisional Biopsies. J. Pers. Med..

[18] Rao R.S., Shivanna D.B., Mahadevpur K.S., Shivaramegowda S.G., Prakash S., Lakshminarayana S., Patil S. (2021). Deep Learning-Based Microscopic Diagnosis of Odontogenic Keratocysts and Non-Keratocysts in Haematoxylin and Eosin-Stained Incisional Biopsies. Diagnostics.

